# Divergent microbiome signatures between managed and wild honey bee (*Apis mellifera*) populations in South Texas

**DOI:** 10.1128/spectrum.03394-25

**Published:** 2026-01-29

**Authors:** Erick V. S. Motta, Jane Seong, Myra Dickey, Jordan T. Ellis, Juliana Rangel

**Affiliations:** 1 Department of Entomology, Texas A&M University 14736, College Station, Texas, USA; University of Florida, Gainesville, Florida, USA

**Keywords:** honey bee, gut microbiome, microbial diversity, wild populations

## Abstract

Animal microbiomes are shaped by a complex interplay of host genetics, environment, and lifestyle. Transitions of animals from wild to managed or domesticated conditions can impact the structure and function of their microbiomes. Among insects, the Western honey bee (*Apis mellifera*) is a promising model for studying these dynamics, given its long history of domestication and importance in agriculture. Despite extensive research on managed honey bee microbiomes, direct comparisons with wild populations remain scarce. Here, we compared the gut microbiomes of wild honey bee colonies from the Welder Wildlife Refuge in South Texas, which have remained unmanaged for over 30 years, with those from the closest managed apiary, located approximately 40 miles away. Using quantitative PCR and 16S ribosomal RNA gene sequencing, we found that managed bees harbored significantly higher bacterial loads but lower microbial diversity than wild bees. Although both groups shared a conserved microbiome dominated by core bacterial genera, distinct amplicon sequence variants and predicted metabolic pathway profiles differentiated them. Managed bee microbiomes were enriched in amino acid interconversion and antibiotic resistance-associated pathways, whereas wild bee microbiomes exhibited broader metabolic versatility, including amino acid biosynthesis and aromatic compound degradation. PCR screening further revealed a higher prevalence of tetracycline resistance markers in managed bees. Together, these findings indicate that long-term absence of human management and distinct ecological pressures shape honey bee gut microbiomes, influencing community composition and potentially functional capacity, underscoring the importance of natural environments to maintain microbial diversity and resilience.

**IMPORTANCE:**

Understanding how human management shapes the microbiomes of domesticated species is essential for predicting their resilience to environmental stressors. Honey bees (*Apis mellifera*) are critical pollinators whose health and productivity are closely tied to their gut microbiota, but nearly all microbiome studies have focused on managed colonies. This study leverages the Welder Wildlife Refuge in South Texas, one of the few documented and well-studied sites in the United States where honey bee colonies have persisted unmanaged for decades, to directly compare managed and wild populations. We show that managed bees harbor higher bacterial loads but reduced microbial diversity and greater antibiotic resistance potential. These findings demonstrate that long-term absence of human intervention can preserve microbial diversity, offering insights into how domestication, antibiotic exposure, and environmental simplification influence the evolution and ecology of host-associated microbiomes.

## INTRODUCTION

The transition from wild to managed conditions often drives profound changes in animal populations, influencing their physiology, behavior, and interactions with the environment (1, 2). Human management, whether through captivity, selective breeding, or controlled diets, can introduce novel stressors and disrupt natural ecological dynamics (3). Over time, these pressures may lead to altered developmental trajectories, reduced genetic diversity, and modified disease resistance in managed populations compared to their wild counterparts (4, 5). Among the most sensitive indicators of such changes are the gut microbiomes, which are complex microbial communities shaped by host genetics, diet, social environment, and ecological context (6). In many animal species, the gut microbiome plays a central role in digestion, immunity, and overall health, and for that reason, it has emerged as a key marker of physiological divergence between wild and managed populations, as well as a promising system for understanding how human intervention reshapes animal biology over time (7–12).

The honey bee (*Apis mellifera*) is one of the most extensively managed insect species worldwide, with colonies being maintained in artificial hives for commercial pollination and honey production across diverse landscapes (13). Beekeeping dates back over 9,000 years, with human-honey bee interactions being documented in early civilizations such as Egypt and Mesopotamia (14). Genetic evidence supports an origin of *A. mellifera* in Africa or the Middle East, followed by at least two ancient, natural migrations into Europe prior to domestication (15, 16). Subsequent human introductions spread managed populations to all continents except Antarctica (15).

Despite this long history of management and global expansion, *A. mellifera* harbors a remarkably specialized gut microbiome dominated by five to nine bacterial genera that together comprise over 90% of the gut community. This includes a core consistently found in nearly all adult honey bees (*Snodgrassella*, *Gilliamella*, *Lactobacillus* Firm-5, *Bombilactobacillus*, and *Bifidobacterium*), as well as others that are variably present (*Bartonella*, *Frischella*, *Commensalibacter*, and *Bombella*) (17, 18). These core bacteria are socially transmitted shortly after adult emergence through contact with nurse bees and hive materials, establishing a stable and host-adapted microbiome that evolved with its honey bee host (19). Experimental studies have shown that the honey bee gut microbiome contributes to digestion, development, and pathogen resistance (17), and it may also be a reservoir for antimicrobial resistance genes, particularly considering the long-term use of antibiotics in beekeeping (20).

**Fig 1:**
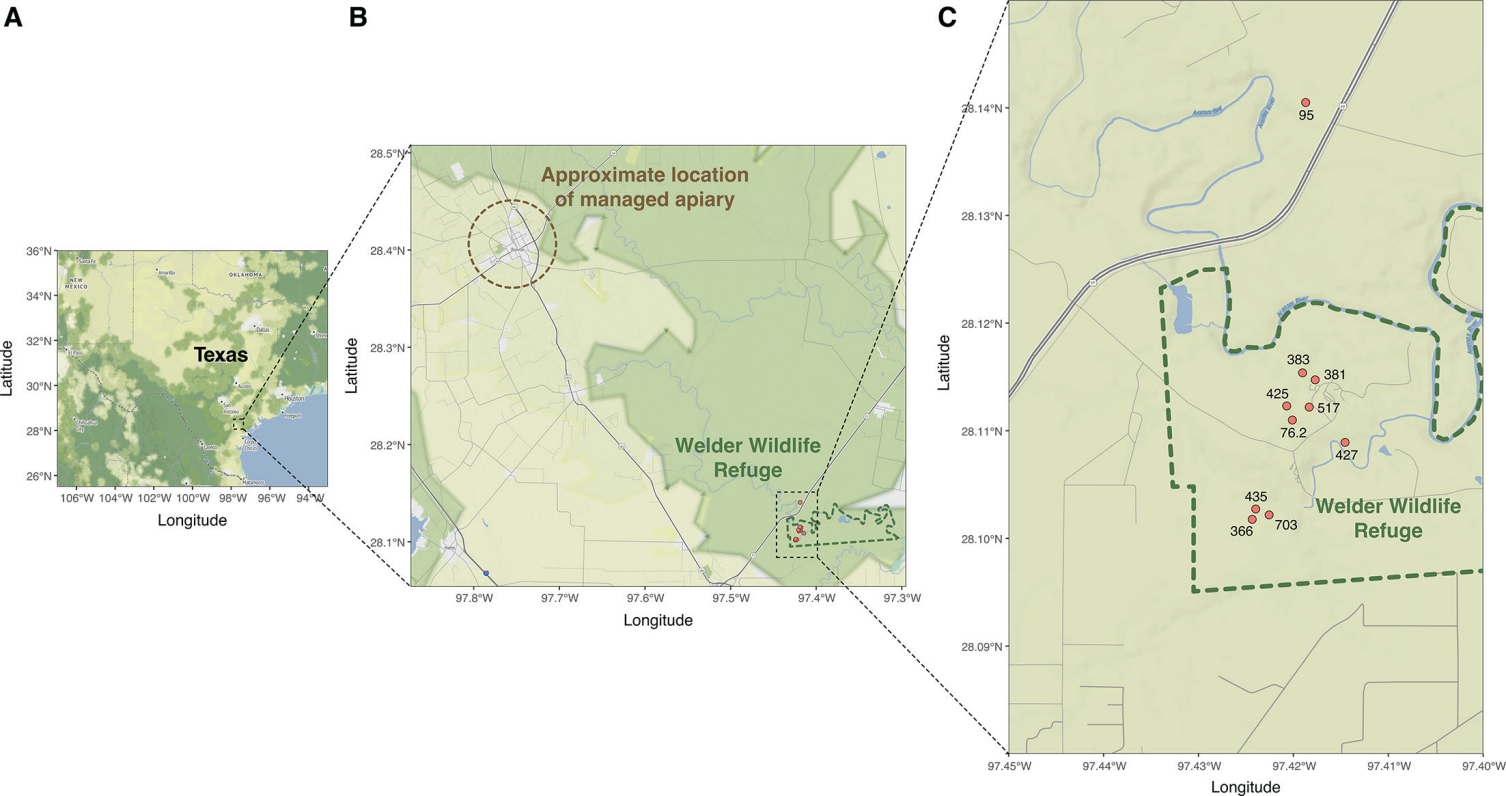
Sampling locations of managed and wild honey bee colonies in South Texas. (**A**) Map of Texas with a dashed square marking the region where all sampling occurred. (**B**) Zoomed map showing the locations of the managed apiary and the WWR and their spatial separation. The managed apiary is shown at a generalized position, and a dashed brown 5 km radius circle is drawn around it to mask exact coordinates for landowner privacy. The WWR boundary was delimited using the protected areas database of the United States (PAD-US, USGS) and is displayed as a dashed green boundary. (**C**) Further zoom into the WWR highlighting the exact positions of sampled wild colonies. Maps were generated in R using ggmap and ggplot2. Basemap tiles are from Stadia Maps (“stamen_terrain” style), and state boundaries and city labels are from the maps package.

While gut microbiomes of managed honey bees have been widely studied (21, 22), comparable research on wild colonies remains scarce. Investigating the microbiomes of wild honey bees offers a unique opportunity to understand how long-term absence of human management, natural environmental pressures, and evolutionary history shape host-microbial interactions, potentially revealing differences in microbial community composition, diversity, and functional capacity compared to managed populations. The Welder Wildlife Refuge (WWR), located in South Texas, United States, hosts a long‐established population of wild honey bee colonies that have lived unmanaged for over 30 years approximately 40 miles from the nearest managed apiaries (23, 24) (Fig. 1). During this time, several studies have revealed unique patterns in the population genetics and disease ecology of colonies at the WWR, including a shift from exclusively European maternal lineages (mitotypes) in the 1990s to over 90% Africanized A-lineage mitotypes by the 2000s, as well as the detection of RNA viruses and *Nosema* at low to medium intensity levels (23–27). However, despite those extensive studies, the gut microbiome of these wild honey bee colonies has not yet been characterized.

In this study, we investigated and compared the gut microbiomes of wild honey bee populations sampled from the WWR to those from the closest managed apiary to test whether management and domestication were associated with changes in these populations’ gut microbial community composition. We extracted DNA from gut samples and performed quantitative PCR (qPCR) to investigate microbiome abundance and 16S ribosomal RNA (rRNA) gene sequencing to characterize community composition and diversity. Our results will help us elucidate whether long-term absence of human management and ecological divergence at the WWR are associated with distinct gut microbiomes in wild honey bees.

## MATERIALS AND METHODS

### Study site

This study builds on samples collected from a previous survey conducted at the WWR in San Patricio County, Texas (28°06′53″, −97°25′01″) (Fig. 1), as reported in (24). The refuge spans a transitional zone where the South Texas Plains, Gulf Prairies, and marsh ecoregions meet. The vegetation includes live oaks, mesquites, brushland, and grasslands, and the region typically experiences humid conditions with hot summers and cool winters (28).

### Sample collection

Wild honey bee foragers were collected by netting in August 2021 from the entrances of naturally occurring tree cavities at the WWR, where active colonies had been identified and tagged in earlier surveys (24). Samples were immediately preserved on dry ice and were subsequently stored at −80°C until processing. Managed honey bee foragers were collected by netting in August 2021 from hive entrances at a stationary apiary located approximately 40 miles from the WWR. The exact coordinates of this apiary cannot be provided due to landowner privacy and are therefore shown only as a generalized position, with the true location lying within the 5 km radius depicted in Fig. 1. The beekeeper maintained around 100 colonies for queen rearing and honey production using standard apicultural practices, including *Varroa* mite treatment and requeening. Samples were also immediately preserved on dry ice and were subsequently stored at −80°C until processing. We randomly selected 5 workers from each of 10 wild colonies and 10 managed colonies for microbiome analysis.

### DNA extraction

DNA was extracted from individual guts of managed bees (*n* = 5 workers per colony, 10 colonies total) and wild bees (*n* = 5 workers per colony, 10 colonies total) using a previously described protocol (29), with some modifications. Guts were homogenized in 100 μL of cetyltrimethylammonium bromide (CTAB) buffer (0.1 M of Tris-HCl pH 8.0, 1.4 M of NaCl, 20 mM of EDTA, and 2% [wt/vol] of CTAB) and then resuspended in an additional 600 μL of CTAB buffer and 20 μL of proteinase K solution (0.1 M Tris-HCl, 26 mM CaCl_2_, 50% glycerol, and 20 mg/mL proteinase K). Each sample was transferred to a capped vial containing 0.5 mL of autoclaved 0.1 mm Zirconia beads (BioSpec Products). Next, 2 μL of 2-b-mercaptoethanol was added, and samples were bead-beaten twice for 2 min each. Samples were then digested at 56°C overnight, transferred to a 2 mL microtube, and mixed with 500 μL of phenol-chloroform-isoamyl alcohol (25:24:1, pH 8.0). The mixture was inverted five times and centrifuged for 15 min at 4°C and 18,000 relative centrifugal force (RCF). The upper aqueous layer was transferred to a new 1.5 mL microtube, followed by the addition of 500 μL of isopropanol and 50 μL of 3 M sodium acetate (pH 5.4). Samples were incubated at −20°C overnight to precipitate the DNA. The following day, samples were centrifuged for 30 min at 4°C and 18,000 RCF, and the supernatant was removed. DNA pellets were washed with 1 mL of ice-cold 75% ethanol and centrifuged for an additional 3 min at 4°C. After removing the ethanol wash, pellets were air-dried at room temperature for 30 min and resuspended in 50 μL of nuclease-free water. DNA samples were stored at −20°C.

### qPCR analysis

DNA samples were diluted 10-fold for downstream analyses. For qPCR, triplicate reactions were prepared in 96-well plates and run on a Bio-Rad CFX384 Touch Real-Time PCR instrument. Each reaction contained 5 μL of iTaq Universal SYBR Green Supermix (BioRad), 0.05 μL of 100 μM forward (27F: 5′-agagtttgatcctggctcag-3′) and reverse (355R: 5′-ctgctgcctcccgtaggagt-3′) primers, 3.9 μL of water, and 1.0 μL of template DNA. Cycling conditions included an initial step at 50°C for 2 min and 95°C for 2 min, followed by 40 cycles of 95°C for 15 s and 60°C for 1 min.

Total bacterial 16S rRNA gene copies were quantified using a standard curve generated from a synthesized 347 bp 16S rRNA gene fragment (Twist Bioscience). The fragment represents the 16S rRNA gene region amplified from *Escherichia coli* K12 covered by the 27F and 355R primers. The fragment concentration was quantified (Qubit) and adjusted to 10^10^ copies/μL. Then, serial dilutions were prepared from 10^8^ to 10^2^ copies/μL and used as standards and run in triplicate. The resulting standard curve showed an amplification efficiency of 91.34% and an *R*^2^ of 0.9991. 16S rRNA gene copy numbers were calculated as 10^(Ct-b)/m^, where “b” and “m” are the *y*-intercept and slope of the standard curve, respectively, and cycle threshold (Ct) was the average of triplicates. Values were corrected for the dilution factor.

Absolute bacterial abundance (16S rRNA gene copies per gut) was analyzed using a linear mixed-effects model fitted with the lmer function from the R package lmerTest (30). The response variable (qPCR counts) was log_10_-transformed qPCR counts to meet model assumptions of normality and homoscedasticity. The model included Group (Managed vs Wild) as a fixed effect and Colony as a random effect to account for non-independence among samples from the same colony. Model significance was assessed using Satterthwaite’s method for degrees of freedom and *t*-test.

### 16S rRNA gene sequencing

Ten-fold diluted DNA samples were also used as templates for 16S rRNA gene library preparation. An initial PCR reaction was performed to amplify the V4 region of the 16S rRNA gene using 1 μL of 10 μM forward and reverse primers (Hyb515F: 5′-tcgtcggcagcgtcagatgtgtataagagacaggtgycagcmgccgcggta-3′, and Hyb806R: 5′-gtctcgtgggctcggagatgtgtataagagacagggactachvgggtwtctaat-3′) (31), 12.5 μL of AccuStart II PCR SuperMix (Quantabio), 8.5 μL of water, and 2 μL of template DNA. Cycling conditions consisted of 94°C for 3 min, 30 cycles of 94°C for 20 s, 50°C for 15 s, 72°C for 30 s, and 72°C for 10 min.

A second PCR was then performed to attach dual indices and Illumina sequencing adapters to the products from the first PCR reaction, including a unique combination of 2 μL of 5 μM index primers (Hyb-F*nn*-i5: 5′-aatgatacggcgaccaccgagatctacacnnnnnntcgtcggcagcgtc-3′, and Hyb-R*nn*-i7: 5′-caagcagaagacggcatacgagatnnnnnngtctcgtgggctcgg-3′) (31), 12.5 μL of AccuStart II PCR SuperMix, 3.5 μL of water, and 5 μL of product from the first PCR reaction. Cycling conditions consisted of 94°C for 3 min, 10 cycles of 94°C for 20 s, 50°C for 15 s, 72°C for 60 s, and 72°C for 10 min.

PCR products were purified using 0.8× HighPrep PCR magnetic beads (MagBio) and quantified by spectrophotometry using a NanoQuant plate on a Tecan Infinite M Plex plate reader. Samples (150 ng each) were pooled and submitted for Illumina sequencing on the MiSeq platform (2 × 250 bp run) at Texas A&M University’s Genomics and Bioinformatics Service (College Station, TX).

### 16S rRNA gene analysis

Illumina sequence reads were demultiplexed and then assessed for quality using FastQC (32) and summarized with MultiQC (33). Downstream analysis was conducted in QIIME 2 version 2024.10 (34) on the Grace cluster at Texas A&M University’s High-Performance Research Computing facility. Paired-end FASTQ files were imported in Casava 1.8 paired-end demultiplexed format. Primers targeting the V4 region of the 16S rRNA gene (forward: gtgycagcmgccgcggta; reverse: ggactachvgggtwtctaat) were trimmed using the cutadapt plugin (35), discarding untrimmed and indel-containing reads.

Denoising, paired-end read merging, quality filtering, and chimera removal were performed using DADA2 with forward and reverse reads truncated at 220 bp (36). Taxonomy was assigned to resulting amplicon sequence variants (ASVs) using the SILVA 138 reference database and the feature-classifier plugin (37). ASVs present at very low abundance (<0.1% of the mean sample read count) were filtered out to reduce potential sequencing noise. Mitochondrial and chloroplast reads were removed using the taxa filter-table plugin.

To mitigate inflation of richness caused by intra-genomic variation in 16S rRNA gene copies within species (38), ASVs were clustered *de novo* at 97% similarity using VSEARCH (39). A final ASV table (File 1) was generated to investigate bacterial diversity and exported for data visualization and statistical analysis in R version 4.3.2 (40). A phylogenetic tree was also constructed using MAFFT for sequence alignment (41) and FastTree for tree inference (42) and used for beta diversity analysis.

Bacterial diversity analyses were performed using the R package phyloseq (43). Data (ASV table, phylogenetic tree, and mapping file) were imported and merged into a single phyloseq object. The distribution of sequencing depths across samples was examined using rarefaction curves to identify an appropriate rarefaction depth (Fig. S1). Based on this assessment, read counts were rarefied to an even depth of 3,616 reads per sample using a custom function (44), ensuring comparable diversity coverage across samples while retaining the full data set. For alpha diversity, which measures taxonomic diversity within samples, the number of observed ASVs, the Shannon diversity index, and the Simpson index were calculated. To assess differences between groups, linear mixed-effects models were fitted for each alpha diversity metric using the lmer function from the R package lmerTest (30), with Group (Managed and Wild) as a fixed effect and Colony as a random effect. Model significance was assessed using Satterthwaite’s method for degrees of freedom and *t*-tests. To quantify the magnitude of group differences, Cohen’s *d* was calculated for each alpha diversity metric using cohens_d function in the R package effectsize (45), using the formula *d* = (Mean_Managed − Mean_Wild) / SD_Pooled, where the numerator represents the difference in group means and the denominator is the pooled standard deviation. For beta diversity, which measures taxonomic diversity between samples, ordinations and distance matrices were calculated using the Bray-Curtis and weighted UniFrac metrics and were visualized with non-metric multidimensional scaling (NMDS) plots. Differences between groups were tested using permutational multivariate analysis of variance (PERMANOVA) and the adonis2 function from the R package vegan (46).

Presence and absence patterns of ASVs across groups were assessed by generating a binary matrix indicating ASV presence within each group. Group-specific and shared ASVs were identified, and compositional overlap was visualized using proportional Venn diagrams with the ggvenn and eulerr packages (47, 48). Moreover, differential abundance analysis was conducted using the R package DESeq2 (49). DESeq2 was applied to unrarefied ASV counts, as recommended for negative binomial modeling, and size factors were estimated using the “poscounts” method to account for differences in sequencing depth. Differential abundance was then tested by fitting negative binomial generalized linear models to these DESeq2 normalized counts. Significant differentially abundant ASVs between groups were identified based on adjusted *P*-values (Benjamini-Hochberg correction) and visualized using ggplot2 (50).

### Phylogenetic analysis and taxonomic refinement

A phylogenetic analysis based on the V4 region of the 16S rRNA gene was conducted to refine the taxonomy of ASVs identified as *Bombilactobacillus* or *Lactobacillus*. Reference sequences from strains isolated from honey bees and bumble bees, with available genomes archived in the National Center for Biotechnology Information (NCBI), were included alongside ASVs from this study. Sequences were aligned using MUSCLE (51), trimmed to the V4 region, and used to infer a maximum-likelihood phylogeny (LG model + Gamma4, 1,000 bootstrap replicates) with PhyML 3.1 (52) implemented in SeaView version 5.0.5 (53). This analysis was carried out primarily to clarify the placement of one abundant but ambiguously classified ASV. To complement the phylogenetic approach and provide a conservative interpretation for this ambiguous ASV, a Lowest Common Ancestor (LCA) analysis was performed. The representative ASV sequence was queried against the NCBI curated 16S rRNA database using BLASTN v2.16.0+ (54) with the parameters -max_target_seqs 50, -max_hsps 1, and -evalue 1e-20. Accession numbers from the top 50 high-scoring pairs were extracted from BLAST XML output and assigned taxonomy using the taxonomizr R package v0.11.1 (55), which maps nucleotide accessions to NCBI Taxonomy identifiers. Full lineage information was retrieved for each hit, and the LCA was computed by identifying the deepest taxonomic rank shared across all non-redundant lineages. This approach allowed determination of the highest reliably supported rank for the ambiguous ASV.

### Inferred metabolic pathway analysis

Microbial metabolic pathway abundances were predicted from 16S rRNA gene sequences using PICRUSt2 version 2.6.2 (56). The resulting data were imported into R version 4.3.2 (40) for downstream analysis using the ggpicrust2 package (57). Differential abundance analysis of predicted pathways was performed using the LinDA method (58), which accounts for the compositional nature of microbiome data and corrects for multiple testing. Pathway annotation was conducted specifying MetaCyc as the pathway database and disabling Kyoto Encyclopedia of Genes and Genomes (KEGG) Orthology to KEGG conversion (ko_to_kegg = FALSE) to retain MetaCyc pathway identities. Comparisons were made between Managed and Wild bee groups as defined in the metadata, with statistical significance being determined at an adjusted *P*-value threshold of 0.05. Significant pathways were manually classified into functional pathway classes based on a curated MetaCyc pathway classification table to facilitate biological interpretation and visualization. For heatmap visualization, pathway abundances corresponding to significant pathways were averaged across samples from the same colony to reduce within-colony variability. The averaged abundance matrix was then standardized using *Z*-score normalization, with missing or infinite values being replaced by zero. Heatmaps were generated using the pheatmap R package.

### PCR screening of antimicrobial resistance markers

Five microliters of 10-fold diluted DNA from individual bees were pooled by colony source for screening eight antimicrobial resistance markers: *tetB*, *tetC*, *tetD*, *tetH*, *tetL*, *tetY*, *tetM*, and *tetW* (Table S1). In total, 10 pooled samples were generated from managed colonies and 10 from wild colonies. Each PCR reaction contained 0.125 μL of Taq DNA Polymerase (New England Biolabs), 2.5 μL of 10× standard Taq reaction buffer, 0.5 μL of 10 mM dNTPs, 0.5 μL of each 10 μM forward and reverse primer (primer sequences are shown in Table S1), 19.875 μL of nuclease-free water, and 1 μL of pooled template DNA. Cycling conditions consisted of an initial denaturation at 95°C for 2 min, followed by 35 cycles of 95°C for 30 s, 60°C for 30 s, and 68°C for 30 s, with a final extension at 68°C for 5 min. PCR products were visualized on 1% agarose gels, with genomic DNA from *Snodgrassella alvi* wkB2 used as a positive control when appropriate.

Presence and absence of each *tet* marker was visualized as a binary heatmap using pheatmap in R and summarized by group as bar plots with overlaid counts using ggplot2 in R. Group differences in prevalence were assessed with two-sided Fisher’s exact tests on 2 × 2 contingency tables, with *P*-values adjusted by the Benjamini-Hochberg false discovery rate (FDR) method. Markers without detection (e.g., *tetD* and *tetM*) were excluded. All analyses were performed in R, with statistical significance set at FDR-adjusted α = 0.05.

## RESULTS

### Managed and wild honey bees differ in gut bacterial loads

**Fig 2:**
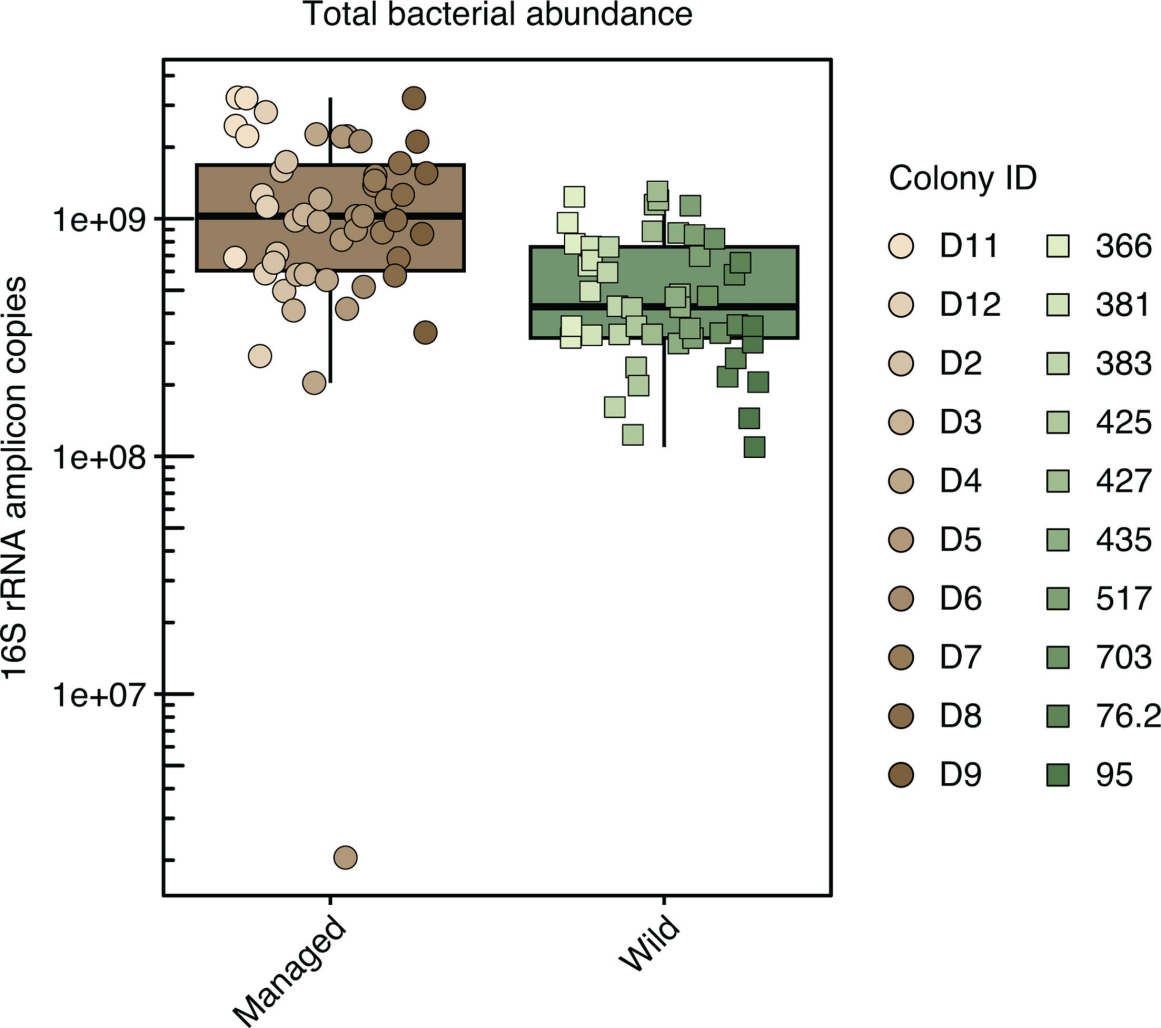
Comparisons of bacterial loads in managed and wild honey bees. The box plot shows estimates of total bacterial abundance in managed (*n* = 50) and wild bee guts (*n* = 50). Statistical analysis was performed using a linear mixed-effects model on log-transformed data (*t*(18.3) = −3.46, *P* = 2.72 × 10^−3^, random effect: Colony).

Estimation of bacterial abundance by qPCR revealed that managed honey bees exhibited a significantly higher average of 16S rRNA gene copies per gut (1.26 × 10^9^, 95% confidence interval [CI]: 1.03 × 10^9^ to 1.48 × 10^9^, *n* = 50) compared to wild honey bees (average = 5.35 × 10^8^, 95% CI: 4.46 × 10^8^ to 6.24 × 10^9^, *n* = 50) (linear mixed-effects model on log-transformed data, *t*(18.3) = −3.46, *P* = 2.72 × 10^−3^, random effect: Colony) (Fig. 2).

### Managed and wild honey bees differ in bacterial diversity profiles

**Fig 3:**
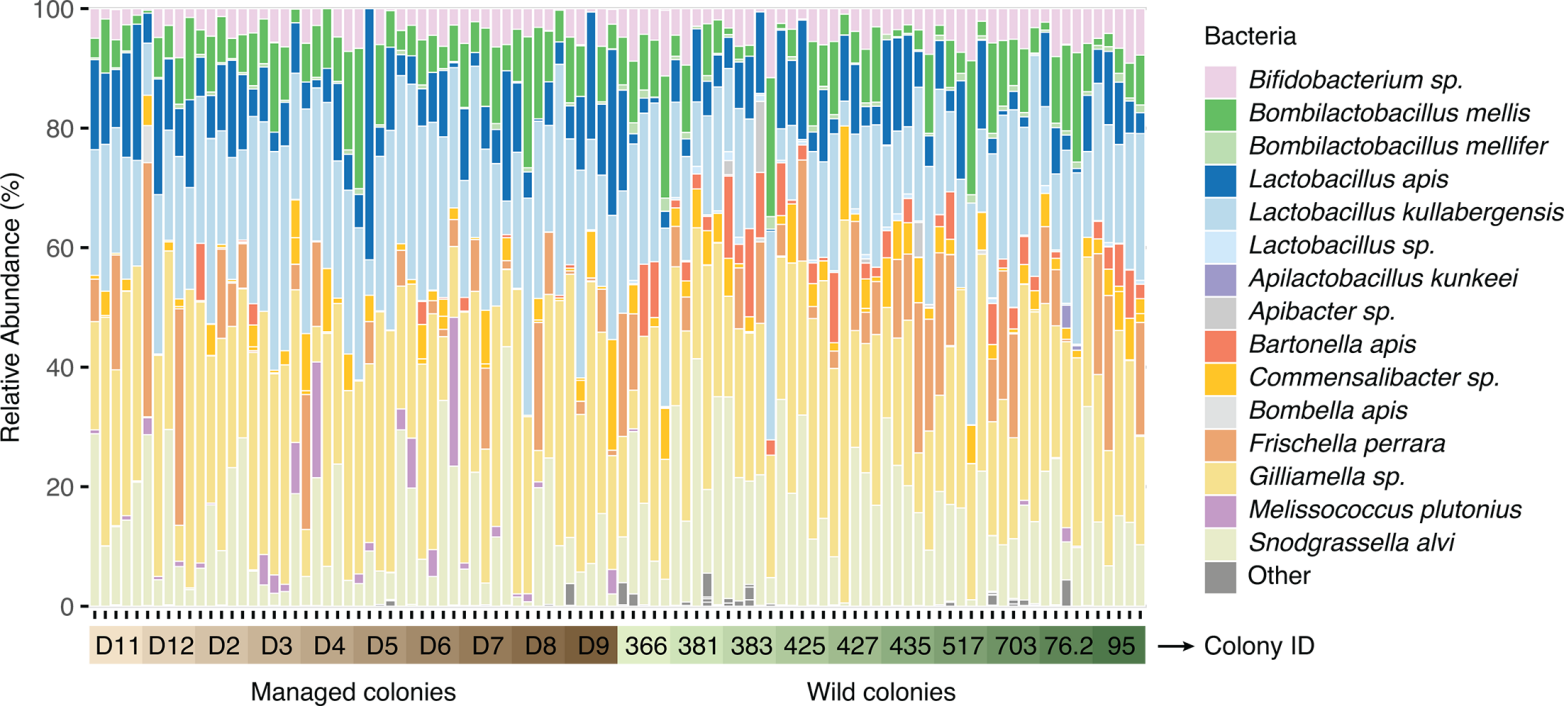
Stacked column graphs showing the relative abundance of bacterial ASVs in managed (*n* = 50) and wild honey bee guts (*n* = 50). Five bees were collected from each colony, with 10 colonies per group.

16S rRNA gene sequencing revealed a similar core microbiota in both managed and wild bees, with *Bifidobacterium*, *Bombilactobacillus*, *Lactobacillus* Firm-5, *Gilliamella*, and *Snodgrassella* dominating the samples (Fig. 3). *Bartonella*, *Commensalibacter*, and *Frischella* were also detected in several samples. Additional genera identified at lower abundances or in a smaller subset of samples included *Apilactobacillus*, *Apibacter*, *Bombella*, and *Melissococcus*. Overall, these patterns align with prior work on the *A. mellifera* gut microbiota (17).

**Fig 4:**
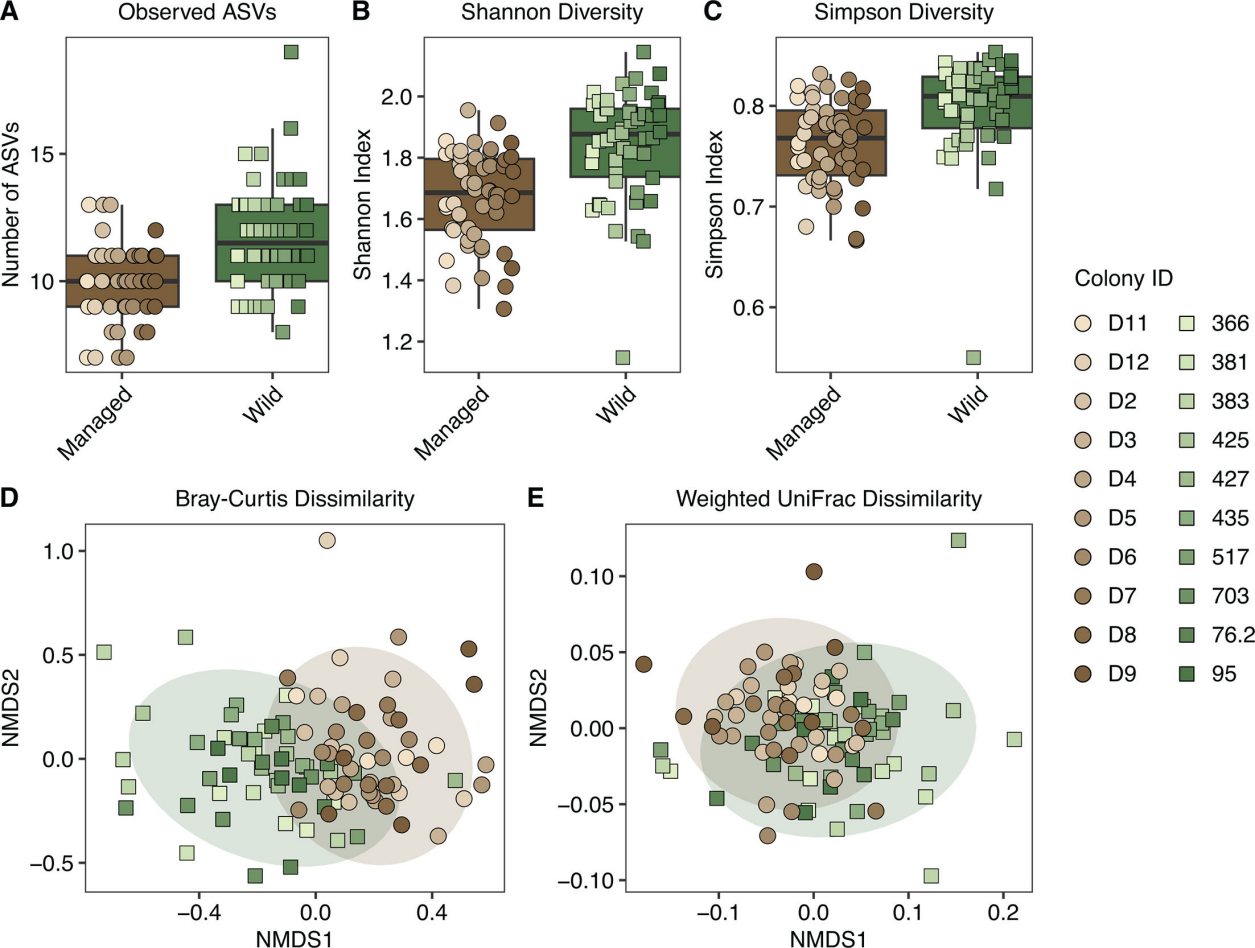
Microbial diversity analysis comparing gut microbiomes in managed and wild honey bees. Alpha diversity was measured as (**A**) the number of observed ASVs (LMM, log-transformed data, *t*(18.0) = 4.09, *P* = 6.92 × 10^−4^), (**B**) Shannon diversity index (LMM, log-transformed data, *t*(18.0) = 5.08, *P* = 7.88 × 10^−5^), and (**C**) Simpson diversity index (LMM, log-transformed data, *t*(*18.0*) = 3.99, *P* = 8.56 × 10^−4^). Beta diversity was assessed using (**D**) Bray-Curtis dissimilarity (PERMANOVA, *F*_1,98_ = 7.32, *P* = 0.001, permutations = 999) and (**E**) weighted UniFrac dissimilarity (PERMANOVA, *F*_1,98_ = 12.54, *P* = 0.001, permutations = 999) and visualized with NMDS plots.

Despite sharing a similar core microbiota, managed and wild bees exhibited distinctly structured gut communities. Wild bees showed significantly greater alpha diversity across all metrics tested (Fig. 4A through C), with linear mixed-effects models confirming higher observed ASVs (*t*(18.0) = 4.09, *P* = 6.92 × 10^−4^), higher Shannon diversity (*t*(18.0) = 5.08, *P* = 7.88 × 10^−5^), and higher Simpson diversity (*t*(18.0) = 3.99, *P* = 8.56 × 10^−4^) in wild bees compared to managed bees. Effect sizes supported these patterns, indicating large differences between groups (Observed ASVs: *d* = −0.90, 95% CI: −1.31 to −0.49; Shannon diversity: *d* = −1.03, 95% CI: −1.44 to −0.61; Simpson diversity: *d* = −0.82, 95% CI: −1.23 to −0.41).

Beta diversity analyses using Bray-Curtis and weighted UniFrac distances revealed significant differences in gut community composition and phylogenetic structure between managed and wild bees (Fig. 4D and E), as supported by our PERMANOVA results (Bray-Curtis: *F*_1,98_ = 7.35, *R*^2^ = 0.07, *P* = 0.001, permutations = 999; weighted UniFrac: F_1,98_ = 12.54, *R*^2^ = 0.11, *P* = 0.001, permutations = 999). These results show that managed and wild honey bees harbor distinct gut microbiome structures, with differences emerging not at the level of core taxa but rather across the broader community composition and phylogenetic organization.

### Managed and wild honey bee microbiomes vary in specific ASVs

We conducted two analyses to identify the ASVs driving bacterial diversity differences between managed and wild honey bees. First, we examined the ASVs found exclusively in either managed or wild honey bees. This analysis identified 22 ASVs present only in wild bees and 15 ASVs present only in managed bees (Fig. S2). Several of these ASVs belonged to the same genera (e.g., *Bartonella*, *Gilliamella*, and *Lactobacillus* Firm-5) and were detected only in one or a few samples, suggesting that unique bacterial species or strains contribute to the observed community differences (Fig. S2).

**Fig 5:**
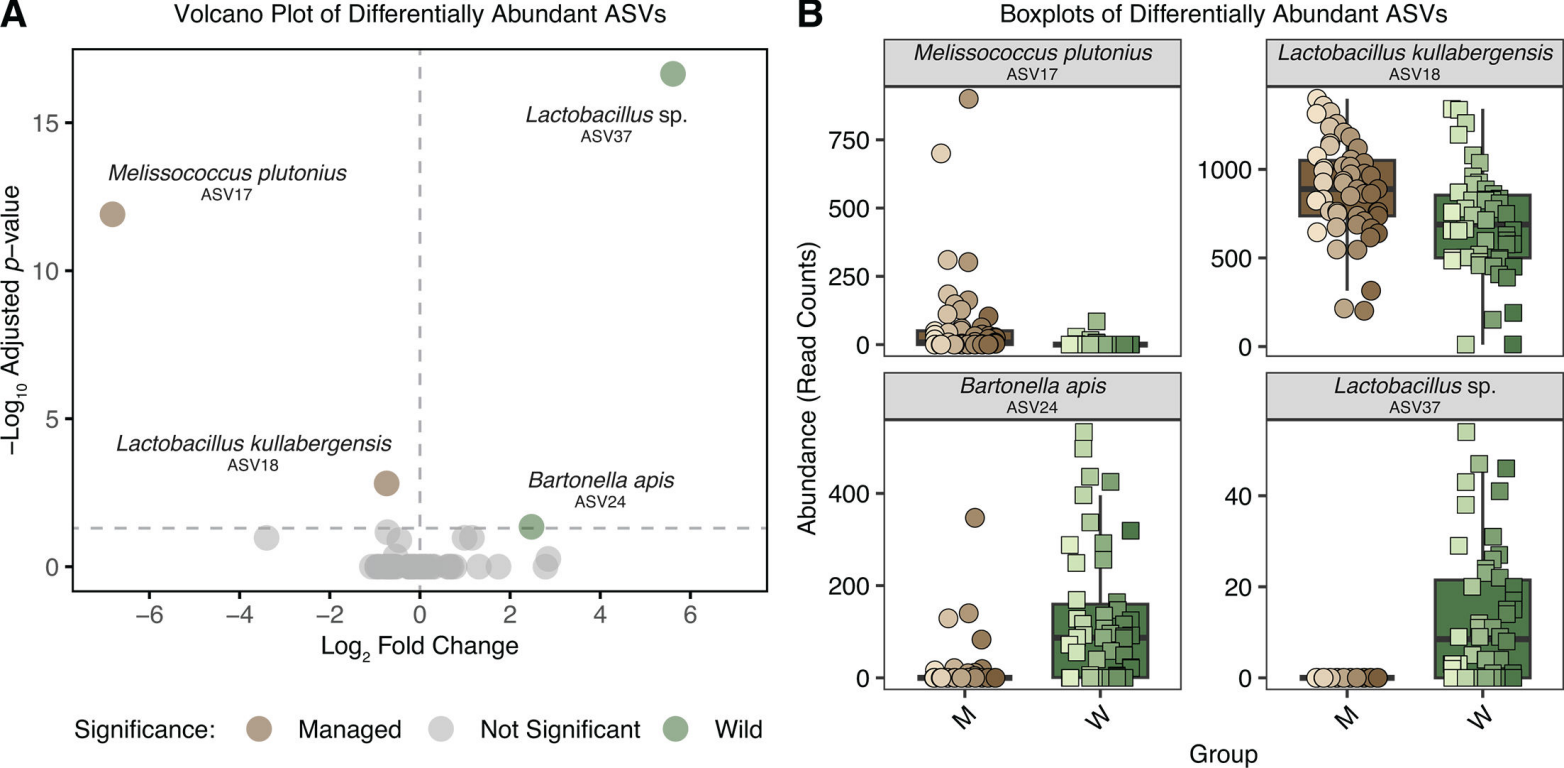
Microbiome differences between managed and wild honey bees. (**A**) Volcano plot showing differential abundance of ASVs between groups. The *x*-axis represents the log_2_ fold change in ASV relative abundance (positive values indicate higher abundance in wild bee guts; negative values indicate higher abundance in managed bee guts), and the *y*-axis represents the -log_10_ of the adjusted *P*-values from DESeq2 analysis. ASVs with adjusted *P*-values < 0.05 were considered significantly more abundant in one group. (**B**) Boxplots showing the abundance of ASVs significantly different between managed and wild bee guts. The *y*-axis represents read counts.

Next, we performed a differential abundance analysis and found only four ASVs that varied significantly between groups (DESeq2: adjusted *P*-value < 0.05, *n* = 100). *Bartonella apis* ASV24 and *Lactobacillus* sp. ASV37 were more abundant in wild bees, whereas *Melissococcus plutonius* ASV17 and *Lactobacillus kullabergensis* ASV18 were enriched in managed bees (Fig. 5; Fig. S3). Notably, *Lactobacillus* sp. ASV37 was detected exclusively in wild bees, while *M. plutonius* ASV17 was largely restricted to managed bees, occurring in only 4% of wild samples. Taken together, these differences in the presence or absence of ASVs, as well as their relative abundance, reveal distinct microbial signatures in the gut microbiome of managed and wild honey bee colonies.

### A closer look into *Lactobacillus* and *Bombilactobacillus* ASVs

Taxonomic assignment of Lactobacillaceae in honey bee microbiomes is often limited by the resolution of the 16S rRNA V4 region. In our data set, several ASVs could be resolved to specific species within *Lactobacillus* Firm-5 (i.e., ASVs 6, 14, 18, 19, 49, 54, and 63) and *Bombilactobacillus* (i.e., ASVs 2, 22, 28, and 36). The remaining ASVs, however, could not be classified beyond the genus level. ASVs 7, 12, 16, 37, 46, and 58 were labeled only as *Lactobacillus* sp., and all but ASV37 were deemed rare.

ASV37 was the only unresolved Lactobacillaceae ASV consistently associated with wild honey bees, occurring in 36 of 50 samples (72%). When we attempted to refine its classification using phylogenetic analysis of V4 sequences from reference *Lactobacillus* and *Bombilactobacillus* genomes, ASV37 clustered with *Bombilactobacillus*-like sequences (Fig. S4). However, LCA analysis of BLAST hits did not support a confident genus-level assignment (Table S2), so ASV37 is best interpreted as a Lactobacillaceae-related ASV that is enriched in wild colonies. Interestingly, native bee species have also been shown to harbor diverse Lactobacillaceae distinct from those found in managed honey bees (59, 60), suggesting that ASV37 may represent a wild honey bee-associated lineage within this family.

### Managed and wild honey bee microbiomes exhibit distinct predicted metabolic pathway profiles

PICRUSt-based predictions identified broad differences in the relative abundance of metabolic pathway categories between managed and wild honey bees (Fig. S5 and S6). Managed bees showed higher predicted abundance of pathways related to amino acid interconversion, carbohydrate metabolism, and cell wall biosynthesis, including the peptidoglycan biosynthesis V pathway associated with β-lactam resistance. In contrast, wild bees were enriched in pathways associated with amino acid biosynthesis, aromatic compound degradation, cofactor and vitamin biosynthesis, and diverse energy metabolism pathways. These patterns suggest differences in metabolic emphasis between groups.

### Managed honey bee microbiomes harbor more antimicrobial resistance markers

**Fig 6:**
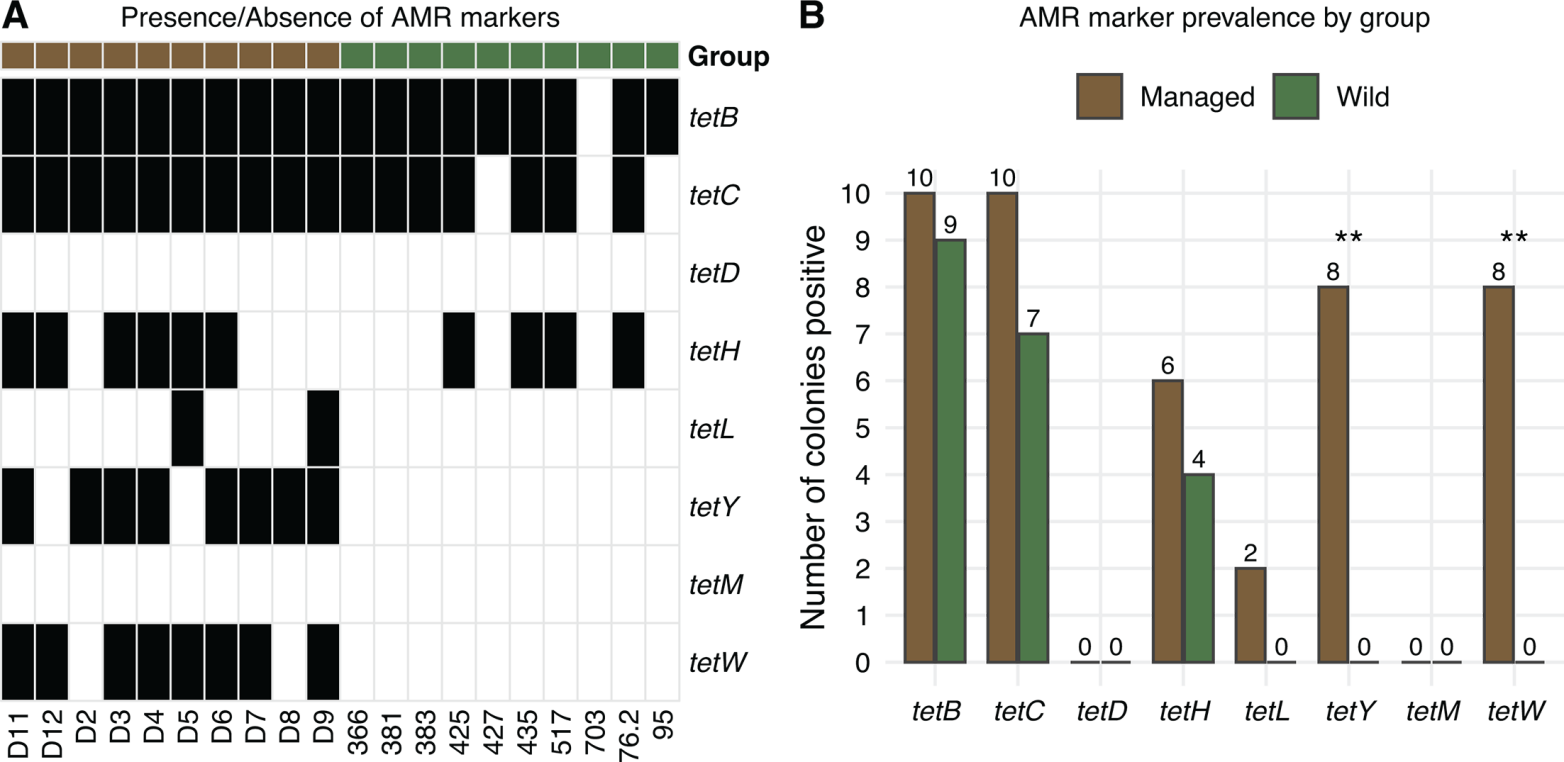
Presence and prevalence of antimicrobial resistance (AMR) markers in managed and wild honey bee colonies. (**A**) Heatmap showing presence (black) or absence (white) of eight tetracycline resistance markers (*tetB*, *tetC*, *tetD*, *tetH*, *tetL*, *tetY*, *tetM*, and *tetW*) across individual colonies. Group identity is indicated by the color bar above (brown = managed, green = wild). (**B**) Prevalence of each AMR marker by group, expressed as the number of positive colonies (out of 10). Bars show managed (brown) and wild (green) colonies, with counts overlaid. Significance was assessed using two-sided Fisher’s exact tests with Benjamini-Hochberg FDR correction (*P* < 0.01, **).

PCR screening revealed a higher prevalence of tetracycline resistance markers in managed colonies compared to wild colonies (Fig. 6). Among wild colonies, nine tested positive for *tetB*, seven tested positive for *tetC*, and four tested positive for *tetH*, while no colonies carried *tetD* or *tetM*. In contrast, all 10 managed colonies tested positive for both *tetB* and *tetC*, 6 for *tetH*, 2 for *tetL*, 8 for *tetY*, and 8 for *tetW* (Fig. 6A). Fisher’s exact tests confirmed significantly higher prevalence of *tetY* and *tetW* (adjusted *P*-value < 0.01) in managed colonies (Fig. 6B). Collectively, these results demonstrate that managed honey bee microbiomes harbor a broader and more frequent repertoire of antimicrobial resistance markers than their wild counterparts, which is consistent with the stronger antibiotic selection that is often observed in managed populations.

## DISCUSSION

This study provides insight into how human management may influence gut microbial communities in Western honey bees by comparing wild colonies from the WWR to managed colonies located approximately 40 miles away. Although both groups shared a consistent core set of dominant gut bacterial genera, they differed in overall bacterial load, alpha and beta diversity, the presence and relative abundance of specific ASVs, and antimicrobial resistance markers. These findings align with growing evidence suggesting that domestication and management can shape animal-associated microbiomes (61, 62). The WWR offers a valuable opportunity to study honey bee colonies that have likely persisted without direct beekeeping intervention for more than three decades, providing a useful comparison for understanding how ecological conditions may influence microbiome structure.

One consistent pattern found was that managed colonies exhibited higher total 16S rRNA gene copies but lower alpha diversity than wild colonies. Several factors in managed settings, such as supplemental feeding, hive manipulations, high colony density, and potential exposure to antibiotics or pesticides, could contribute to these differences by favoring certain bacterial taxa at the expense of community complexity (20, 63, 64). In contrast, wild honey bees at the WWR forage in a highly diverse and unmanaged habitat, potentially encountering a broader array of environmental microbes that may promote greater microbial richness (28, 65). Similar patterns of reduced diversity and increased microbial loads have been reported in managed bumble bees (66), as well as in domesticated mammals (67) compared to their wild relatives, suggesting that this may be a common pattern across taxa.

Fine-scale taxonomic differences between managed and wild bees at the ASV level helped explain the observed differences in microbial diversity. Some ASVs were detected only in wild bees, including *Lactobacillus* sp. ASV37, as well as environmentally associated taxa, such as *Pseudomonas* sp. ASV23, *Sphingomonas* sp. ASV34, and *Stenotrophomonas* sp. ASV5, suggesting that unmanaged habitats may expose bees to a broader pool of microbes. In contrast, the pathogen *M. plutonius*, the causative agent of European foulbrood (EFB), was found primarily in managed colonies, indicating differences in disease exposure that may have contributed to community variation. Environmental factors such as floral composition and local microhabitats have been shown to influence honey bee microbiomes (68–72), which may help explain the taxonomic differences observed in this study.

To explore potential functional differences associated with these taxonomic patterns, we generated predicted metabolic pathway profiles from 16S rRNA gene data using PICRUSt2 (56). These predictions highlighted broad differences in the relative abundance of pathway categories between groups rather than gains or losses of whole functions. Managed bees showed higher predicted abundances of pathways related to amino acid interconversion, carbohydrate degradation, and peptidoglycan biosynthesis associated with β-lactam resistance, with this last one probably reflecting historical exposure to antibiotics (20). In contrast, wild bees exhibited higher predicted abundances of pathways linked to amino acid biosynthesis, aromatic compound degradation, and cofactor synthesis, which are patterns that could be consistent with broader metabolic versatility in unmanaged environments. Because PICRUSt2 relies on 16S rRNA gene data and reference genomes, these predictions should be interpreted cautiously as coarse, putative trends rather than precise, strain-resolved functional profiles. Additional metagenomic or culture-based approaches would be necessary to confirm these functional differences and evaluate their implications for colony health.

Our screening for antimicrobial resistance markers using targeted PCR found higher prevalence of tetracycline resistance markers (e.g., *tetY* and *tetW*) in managed colonies. This pattern is consistent with long-term antibiotic use in US apiculture, which has selected for a suite of tetracycline-resistance determinants in the honey bee gut microbiota (20, 73). In particular, *tetW* has been directly linked to *Bifidobacterium* isolates, whereas other tetracycline-resistance genes, such as *tetB*, *tetC*, *tetD*, and *tetH*, are associated with other gut taxa, such as *Gilliamella* and *Snodgrassella* (20, 74). Antibiotic exposure is also known to reduce microbiome diversity and promote the expansion of resistant strains, effects that can persist across generations even after treatment ceases (75). The absence or lower frequency of these markers in wild colonies aligns with their long-term lack of antibiotic exposure and reduced opportunities for horizontal transfer of resistance elements.

A notable finding was the detection of ASV17 associated with *M. plutonius* primarily in managed colonies, where it occurred in 60% of samples compared to only 4% of wild colonies. Although our sampling targeted asymptomatic adult bees, *M. plutonius* is known to persist in their guts, even when colonies show no clinical signs (76). The higher prevalence of this pathogen in managed colonies likely reflects beekeeping practices, such as brood transplantation, equipment sharing, and high colony density, which often facilitate disease transmission (77). Historically, *M. plutonius* isolates were susceptible to oxytetracycline, but a recent study has documented an oxytetracycline-resistant field isolate, demonstrating that resistance can emerge under antibiotic pressure (78). Although resistance does not appear widespread, increasing EFB prevalence in managed colonies raises concern that reliance on antibiotic treatments may intensify and further shape the microbial resistome in these environments (79). In contrast, the low abundance of this pathogen in wild colonies may reflect their spatial isolation, lower colony density, and minimal exposure to human-mediated transmission pathways.

In addition to pathogen pressures, the genetic background of these populations is also important to consider. The specific colonies sampled at the WWR were previously genotyped and found to have high rates of Africanized maternal lineages (A-lineage mitotype), whereas most managed colonies carried European maternal lineages (C-lineage mitotype), with only a few showing A-lineage ancestry (24). Interestingly, a recent study comparing African, Africanized, and European honey bee microbiomes found that Africanized bees harbored gut microbiomes that were more similar to European bees than to African bees (80). This suggests that the divergence we observed between wild and managed honey bee microbiomes is unlikely to be explained by host genetics alone and instead reflects environmental and management influences.

In summary, our study provides evidence for significant gut microbial divergence between managed and wild honey bee colonies, even over a relatively short ecological timescale. While the core gut microbiota was conserved, wild honey bees exhibited greater alpha diversity and harbored unique ASVs not found in nearby managed populations. These differences may arise from environmental variation, management practices, or both. However, our study represents a single time point and includes only one managed and one wild site. Future work should replicate this comparison across multiple sites, sampling synchronously, and ideally include other unmanaged honey bee populations with long-term isolation, which may be difficult to find. Expanding this research will be crucial to disentangle the effects of management versus local environment on honey bee microbiomes and understand how domestication influences host-microbe evolution.

## Data Availability

16S rRNA gene sequencing data are available on NCBI BioProject PRJNA1305440. Other data are included in this article and its supplemental material.

## SUPPLEMENTAL MATERIAL

Supplemental figures and tables spectrum.03394-25-s0001.docx Figures S1 to S6, and Tables S1 and S2.

ASM does not own the copyrights to Supplemental Material that may be linked to, or accessed through, an article. The authors have granted ASM a non-exclusive, world-wide license to publish the Supplemental Material files. Please contact the corresponding author directly for reuse.
