# Supplementary material for: Divergent microbiome signatures between managed and wild honey bee (Apis mellifera) populations in South Texas
Source: Microbiol Spectr. 2026 Jan 29;14(3):e03394-25. doi: 10.1128/spectrum.03394-25 (PMC12955401; doi:10.1128/spectrum.03394-25)
Supplement: Supplemental figures and tables — Figures S1 to S6, and Tables S1 and S2. [file spectrum.03394-25-s0001.docx]

**Supplemental Material for:**

**Divergent microbiome signatures between managed and wild honey bee (*Apis mellifera*) populations in South Texas**

Erick V. S. Motta^1*^, Jane Seong^1^, Myra Dickey^1^, Jordan T. Ellis^1^, Juliana Rangel^1^

Affiliation:

^1^Department of Entomology, Texas A&M University, College Station, TX, USA

^*^Correspondence: Erick V. S. Motta (erick.motta@ag.tamu.edu)

**This PDF file includes:**

Figures S1 to S6

Tables S1 to S2

**Figure S1.** Rarefaction curves showing the relationship between sequencing depth and observed ASV richness for each sample (blue lines, n = 100). The vertical dashed red line marks the rarefaction depth of 3,616 reads, which was selected as the subsampling threshold because diversity estimates plateaued at this depth and all samples exceeded this minimum read count.

**Figure S2.** Venn diagram showing the number of unique ASVs detected in managed and wild honey bee guts.

**Figure S3.** Boxplots showing relative abundances of representative bacterial amplicon sequence variants (ASVs) in managed (n = 50) and wild honey bee guts (n = 50). Only ASVs detected in at least three samples were included in the plots.

**Figure S4.** Phylogenetic analysis of *Bombilactobacillus* and *Lactobacillus* Firm-5 ASVs based on the V4 region of the 16S rRNA gene. Reference sequences from strains isolated from honey bees and bumble bees and other strains were aligned with ASVs from this study using MUSCLE. Sequences were trimmed to the V4 region (256 nucleotide length) and a maximum-likelihood phylogeny was inferred using PhyML 3.1 (LG model + Gamma4, 1000 bootstrap replicates) implemented in SeaView v5.0.5.

**Figure S5.** Differential abundance of significant MetaCyc metabolic pathways between managed and wild honey bee colonies. The left panel lists pathway names grouped by their metabolic class. The center **panel** shows the mean relative abundance of each pathway by colony type, with bars colored by management status (Managed: brown; Wild: green). The right panel displays the log_2_ fold change in pathway abundance between managed and wild colonies, with bar lengths representing the magnitude and direction of change (log_2_ fold change < 0 indicates greater abundance in managed bees; log_2_ fold change > 0 indicates greater abundance in wild bees), annotated with adjusted *p*-values to indicate significance.

**Figure S6.** Heatmap of metabolic pathways with significant differential abundance between managed and wild honey bee colonies. The heatmap shows row-wise Z-score normalized relative abundances of MetaCyc metabolic pathways identified as significantly different (adjusted *p* < 0.05) from differential abundance analysis of 16S rRNA amplicon data. Pathway abundances were averaged across samples from each colony and then normalized by Z-score across colonies to highlight relative enrichment patterns. Rows represent metabolic pathways (labeled by functional descriptions), and columns represent individual honey bee colonies grouped by management status (Managed vs. Wild). Hierarchical clustering was applied to rows (pathways) to reveal similarity in abundance profiles, while colony columns were not clustered and are annotated by group color.

**Table S1.** Primers used for PCR screening of antimicrobial resistance markers in honey bee gut microbiomes. Reference source: (Tian et al. 2012)

| Target gene | Primer name | Sequence (5′→3′) | Amplicon size (bp) |
| --- | --- | --- | --- |
| *tetB* | TetB-F  TetB-R | TTGGTTAGGGGCAAGTTTTG  GTAATGGGCCAATAACACCG | 659 |
| *tetC* | TetC-F  TetC-R | CTTGAGAGCCTTCAACCCAG  ATGGTCGTCATCTACCTGCC | 418 |
| *tetD* | TetD-F  TetD-R | GAATGCCTGCACCTTTCTGATG  GGCAATAAATCCGGCGAAAA | 346 |
| *tetH* | TetH-F  TetH-R | GTGATGTGACTCCCGCTAAAAAT  CCAGAACCGCCAAAGACATACC | 407 |
| *tetL* | TetL_121F  TetL_335R  TetL_363F  TetL_994R | CCGGCGAGTACAAACTGGGTGA  GCAGCTGCACCAGCTCCTTGAAT  GCGCCTCTGCGAAAGGTACGC  GCGCGCAACTACAACCATCACGAG | 215  631 |
| *tetY* | TetY-F  TetY-R | ATTTGTACCGGCAGAGCAAAC  GGCGCTGCCGCCATTATGC | 181 |
| *tetM* | TetM-F  TetM-R | ACAGAAAGCTTATTATATAAC  TGGCGTGTCTATGATGTTCAC | 171 |
| *tetW* | TetW-F  TetW-R | GAGAGCCTGCTATATGCCAGC  GGGCGTATCCACAATGTTAAC | 168 |

**Table S2.** Summary of NCBI-BLAST hits and LCA assignment for ASV37.

| **(A)** Lowest Common Ancestor (LCA) summary | | | |
| --- | --- | --- | --- |
| Rank | LCA assignment | | |
| Domain | Bacteria | | |
| Phylum | Bacillota | | |
| Class | Bacilli | | |
| Order | Lactobacillales | | |
| Family | Lactobacillaceae | | |
| Genus | Not resolved (multiple genera detected) | | |
| Species | Not resolved | | |
| **(B)** Genus composition of the top 50 BLAST hits | | | |
| Genus | | Count | Percent |
| *Secundilactobacillus* | | 13 | 26% |
| *Lacticaseibacillus* | | 8 | 16% |
| *Latilactobacillus* | | 7 | 14% |
| *Lentilactobacillus* | | 5 | 10% |
| *Bombilactobacillus* | | 5 | 10% |
| *Paucilactobacillus* | | 4 | 8% |
| *Pediococcus* | | 3 | 6% |
| *Agrilactobacillus* | | 3 | 6% |
| *Companilactobacillus* | | 1 | 2% |
| *Lactiplantibacillus* | | 1 | 2% |
